# A CAD system for pulmonary nodule prediction based on deep three-dimensional convolutional neural networks and ensemble learning

**DOI:** 10.1371/journal.pone.0219369

**Published:** 2019-07-12

**Authors:** Wenkai Huang, Yihao Xue, Yu Wu

**Affiliations:** 1 Center for Research on Leading Technology of Special Equipment, School of Mechanical & Electrical Engineering, Guangzhou University, Guangzhou, P.R. China; 2 School of Mechanical & Electrical Engineering, Guangzhou University, Guangzhou, P.R. China; 3 Laboratory Center, Guangzhou University, Guangzhou, P.R. China; University of Oklahoma, UNITED STATES

## Abstract

**Background:**

Detection of pulmonary nodules is an important aspect of an automatic detection system. Incomputer-aided diagnosis (CAD) systems, the ability to detect pulmonary nodules is highly important, which plays an important role in the diagnosis and early treatment of lung cancer. Currently, the detection of pulmonary nodules depends mainly on doctor experience, which varies. This paper aims to address the challenge of pulmonary nodule detection more effectively.

**Methods:**

A method for detecting pulmonary nodules based on an improved neural network is presented in this paper. Nodules are clusters of tissue with a diameter of 3 mm to 30 mm in the pulmonary parenchyma. Because pulmonary nodules are similar to other lung structures and have a low density, false positive nodules often occur. Thus, our team proposed an improved convolutional neural network (CNN) framework to detect nodules. First, a nonsharpening mask is used to enhance the nodules in computed tomography (CT) images; then, CT images of 512×512 pixels are segmented into smaller images of 96×96 pixels. Second, in the 96×96 pixel images which contain or exclude pulmonary nodules, the plaques corresponding to positive and negative samples are segmented. Third, CT images segmented into 96×96 pixels are down-sampled to 64×64 and 32×32 size respectively. Fourth, an improved fusion neural network structure is constructed that consists of three three-dimensional convolutional neural networks, designated as CNN-1, CNN-2, and CNN-3, to detect false positive pulmonary nodules. The networks’ input sizes are 32×32×32, 64×64×64, and 96×96×96 and include 5, 7, and 9 layers, respectively. Finally, we use the AdaBoost classifier to fuse the results of CNN-1, CNN-2, and CNN-3. We call this new neural network framework the Amalgamated-Convolutional Neural Network (A-CNN) and use it to detect pulmonary nodules.

**Findings:**

Our team trained A-CNN using the LUNA16 and Ali Tianchi datasets and evaluated its performance using the LUNA16 dataset. We discarded nodules less than 5mm in diameter. When the average number of false positives per scan was 0.125 and 0.25, the sensitivity of A-CNN reached as high as 81.7% and 85.1%, respectively.

## Introduction

Lung cancer causes more deaths worldwide than any other type of cancer. In the past 50 years, the incidence of lung cancer has significantly increased; it now ranks first in malignant tumors in males and has become a major threat to life and health. However, when an early diagnosis can be made, the 5-year survival rate increases from 10%–16% to 70%. In lung CT, automatic detection of pulmonary nodules plays an important role in a computer-aided diagnosis (CAD) system. Hawkins et al. [1] determined the predictive value of a CT scan for malignant nodules. Using radio physics, lung cancer screenings can be used to assess the risk of lung cancer development through baseline CT scanning.

Pulmonary nodules develop into tumors through a gradual process. People should pay attention to malignant pulmonary nodules and intervene as soon as possible. When malignant nodules are found, patients can undergo operations and improve their survival rate. Pulmonary nodule detection is a key step in improving the sensitivity of the detection of malignant nodules. This study will improve the accuracy of pulmonary nodule detection by helping doctors to detect pulmonary nodules and judge whether they are benign or malignant.

CAD systems are divided into two subcategories: computer-aided detection (CADe) and computer-aided diagnosis (CADx) systems. A CAD system for detecting pulmonary nodules usually involves four stages: lung segmentation, nodule detection, feature analysis, and false positive elimination [2]. To meet these challenges, many researchers have explored extracting information from pulmonary nodules to improve the sensitivity of nodule detection. In the existing approaches, researchers manually extract the features and properties of nodes in CT images and input them into a classifier or neural network such as a Support Vector Machine (SVM) [3], CNN [4], or fully connected neural network [5] to classify nodules and perform semantic feature analysis [6].

**Fig 1:**
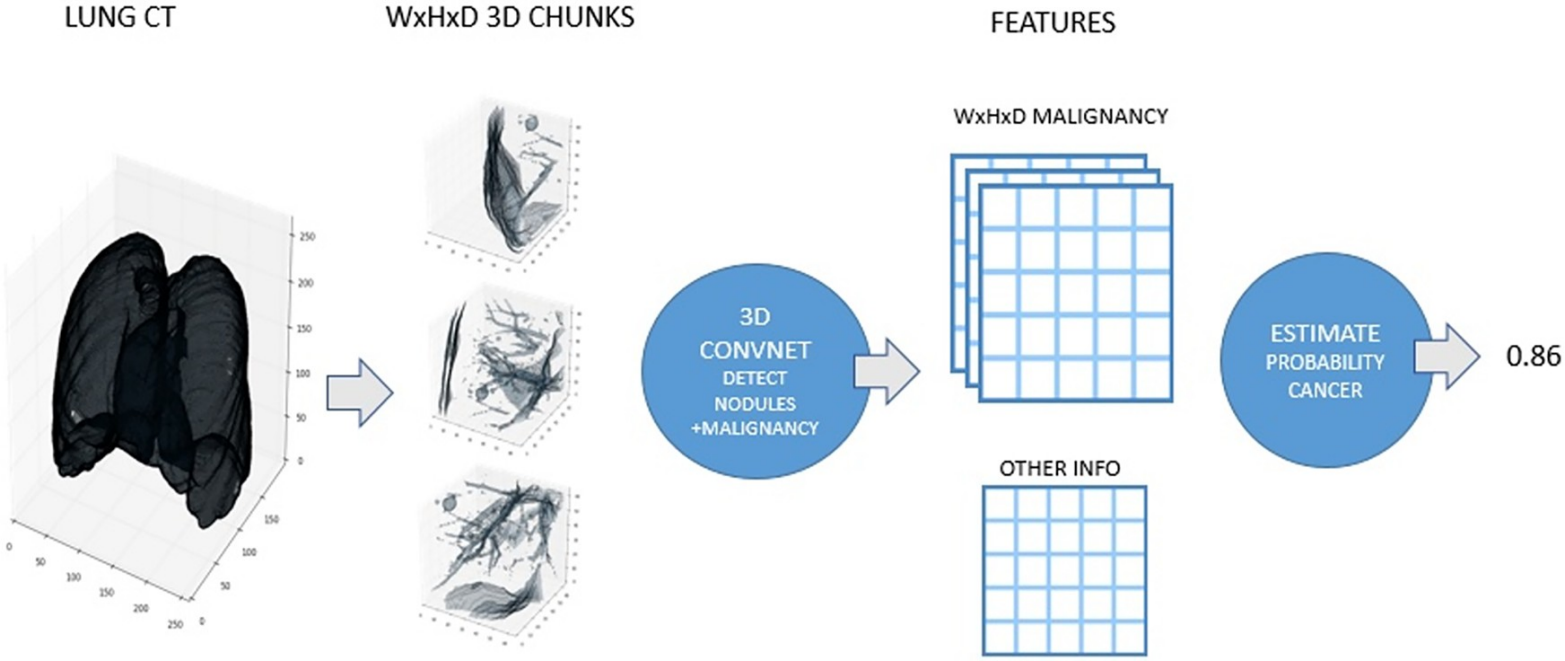
CNN detection of pulmonary nodules.

In recent years, deep learning methods have been introduced into the field of medical image analysis, achieving promising results [7], [8]. CNNs are one of the most popular deep learning methods. In this study, a new neural network is proposed to detect pulmonary nodules that can optimize some of the parameters required to detect pulmonary nodules and improve the sensitivity of the detection steps involved in pulmonary nodule detection ina CAD system. Fig 1 shows a basic CNN flowchart for detecting pulmonary nodules.

Since a CNN first won the ImageNet-Large-Scale-Visual-Recognition-Challenge (ILSVRC)in 2012, they have sparked great achievements in computer vision software applications. In addition, CNNs play an effective role in the field of medical image analysis, especially in detecting nodules and judging their benign vs. malignant qualities.

For nodule detection, Zhao et al. [9] proposed a detection method based onpositron emission tomography (PET) and CT scan data to differentiate malignant from benign pulmonary nodules. Their method combined the characteristics of PET and CT signals and they proposed an improved FP-dimension reduction method to detect pulmonary nodules in PET/CT images using convolutional neural networks. The accuracy of this method reached as high as 95.6%. Shen et al. [10] proposed the Multi-Convolutional Neural Network (MC-CNN) model by adopting a new multicrop aggregation strategy. As different regions are extracted from the convolution feature map, the nodule information is automatically extracted by using maximum pooling at different times. Tajbakhsh et al. [11] compared the performance of two machine learning models, Massive Training Artificial Neural Networks (MTANNs) and CNNs, to detect and classify pulmonary nodules. After using two optimized MTANNs and four different CNN architectures, their experiments showed that MTANNs achieved a better performance. Xia et al. [12] proposed an effective thyroid cancer detection system based on an Extreme Learning Machine (ELM). Due to the characteristics of ultrasound images, the potential of ELM in differentiating benign and malignant thyroid nodules was discussed. The proposed ELM-based method achieved an accuracy of 87.72%, a sensitivity of 86.72%, and a specificity of 94.55%. Xie et al. [13] proposed an automatic detection framework for pulmonary nodules based on a 2D-convolutional neural network. First, two regional networks and one deconvolutional layer were used to adjust the structure of the R-CNN to detect candidate nodules. Then, three different models were trained to obtain the subsequent fused results. Second, an enhanced structure based on 2D-CNN was designed to reduce false positives. Finally, these neural networks were fused to obtain the final classification results. The sensitivity of nodule detection was 86.42%. To reduce the false positives, the sensitivity was 73.4% and 74.4%, respectively, when 1/8 FPs/scan and 1/4 FPs/scan were scanned. Mei et al. [14] proposed using a random forest to predict the malignancy and invasiveness of pulmonary ground-glass nodules (GGNs). The results showed that the malignancy degree of GGNs was 95.1% and the invasive rate of GGNs was 83%, indicating that a random forest model could predict these qualities from lung images. Farahani et al. [15] developed a method to improve the quality of the original CT image based on a type II fuzzy algorithm and proposed a new opportunity-based fuzzy C-means clustering segmentation algorithm to detect nodules. They utilized three classifiers: a multi-layer perceptron (MLP), k-Nearest Neighbor (KNN), and an SVM. Pulmonary nodules were aggregated for benign and malignant diagnoses. Nibali et al. [16] classified pulmonary nodules based on deep residual networks. Based on the latest ResNet architecture, they explored the impacts of curriculum learning, migration learning, and network depth changes on the accuracy of malignant nodule classification. Zhu et al. [17] explored how to tap this vast, but currently unexplored data source to improve pulmonary nodule detection. We propose DeepEM, a novel deep 3D ConvNet framework augmented with expectation-maximization (EM), to mine weakly supervised labels in EMRs for pulmonary nodule detection, respectively, demonstrating the utility of incomplete information in EMRs for improving deep learning algorithms. Wu et al. [18] proposed a deep-neural-network-based detection system for lung nodule detection in CT. A primal-dual-type deep reconstruction network was applied first to convert the raw data to the image space, followed by a 3D-CNN for the nodule detection. With 144 multi-slice fan beam projections, the proposed end-to-end detector could achieve comparable sensitivity with the reference detector, which was trained and applied on the fully-sampled image data. It also demonstrated superior detection performance compared to detectors trained on the reconstructed images. Zhao et al. [19] presented a deep-learning based automatic all size pulmonary nodule detection system by cascading two artificial neural networks. They firstly use a U-net like 3D network to generate nodule candidates from CT images. Then, They use another 3D neural network to refine the locations of the nodule candidates generated from the previous subsystem. With the second sub-system, we bring the nodule candidates closer to the center of the ground truth nodule locations, their system has achieved high sensitivity. Khosravan and Bagci [20] propose S4ND, a new deep learning based method for lung nodule detection. Our approach uses a single feed forward pass of a single network for detection. The whole detection pipeline is designed as a single 3D CNN with dense connections, trained in an end-to-end manner, a high FROC-score have been achieved in the detection of pulmonary nodules. To reduce the incidence of false positive nodules, Zainudin et al. [21]used the deep learning structure of CNN to improve the detection rate of tumors through histopathological images. The performance evaluation of deep learning of CNN was been compared to other classifier algorithm and the result was better accuracy on detecting the mitosis and non-mitosis. Teramoto et al. [22] proposed an improved False Positive (FP)-dimensional reduction method to detect pulmonary nodules in PET/CT images using a CNN. Their FP-dimensional reduction method eliminated 93% of the false positive nodules, and their proposed method used CNN technology to eliminate approximately half of the false positive nodules compared to previous studies. Setio et al. [23] proposed a CADe system that used multi-view convolutional networks (CuNETs) to obtain nodule candidates by combining three candidate detectors specially designed for solid, subsolid, and large nodules. The false-positive nodules among them are then classified along with the false-negative nodules. The accuracy of the section scan reached 90.1%. Cao et al. [24] proposed a new method to compress FP nodules in CT images. First, 87 features were extracted from the candidate nodules, which were divided into five categories (shape, gray level, surface, gradient, and texture) by an SVM and then combined before selecting the most influential features for classification. Then, voxel values were obtained based on MTANNs, and the final result achieved 91.95% accuracy. Suzuki et al. [25]investigated a pattern-recognition technique based on an artificial neural network (ANN), which is called a MTANN, for reduction of false positives in computerized detection of lung nodules in low-dose CT images. By using the Multi-MTANN, the false-positive rate of our current scheme was improved from 0.98 to 0.18 false positives per section (from 27.4 to 4.8 per patient) at an overall sensitivity of 80.3% (57/71). Dou et al. [26] used a new 3D-CNN framework to automatically detect pulmonary nodules, reduce FPs and more effectively distinguish them from their hard simulators. Among the many methods that have been explored, CNNs are most commonly used to detect pulmonary nodules and pseudo positive pulmonary nodules because they can achieve a high sensitivity to reduce FPs.

**Fig 2:**
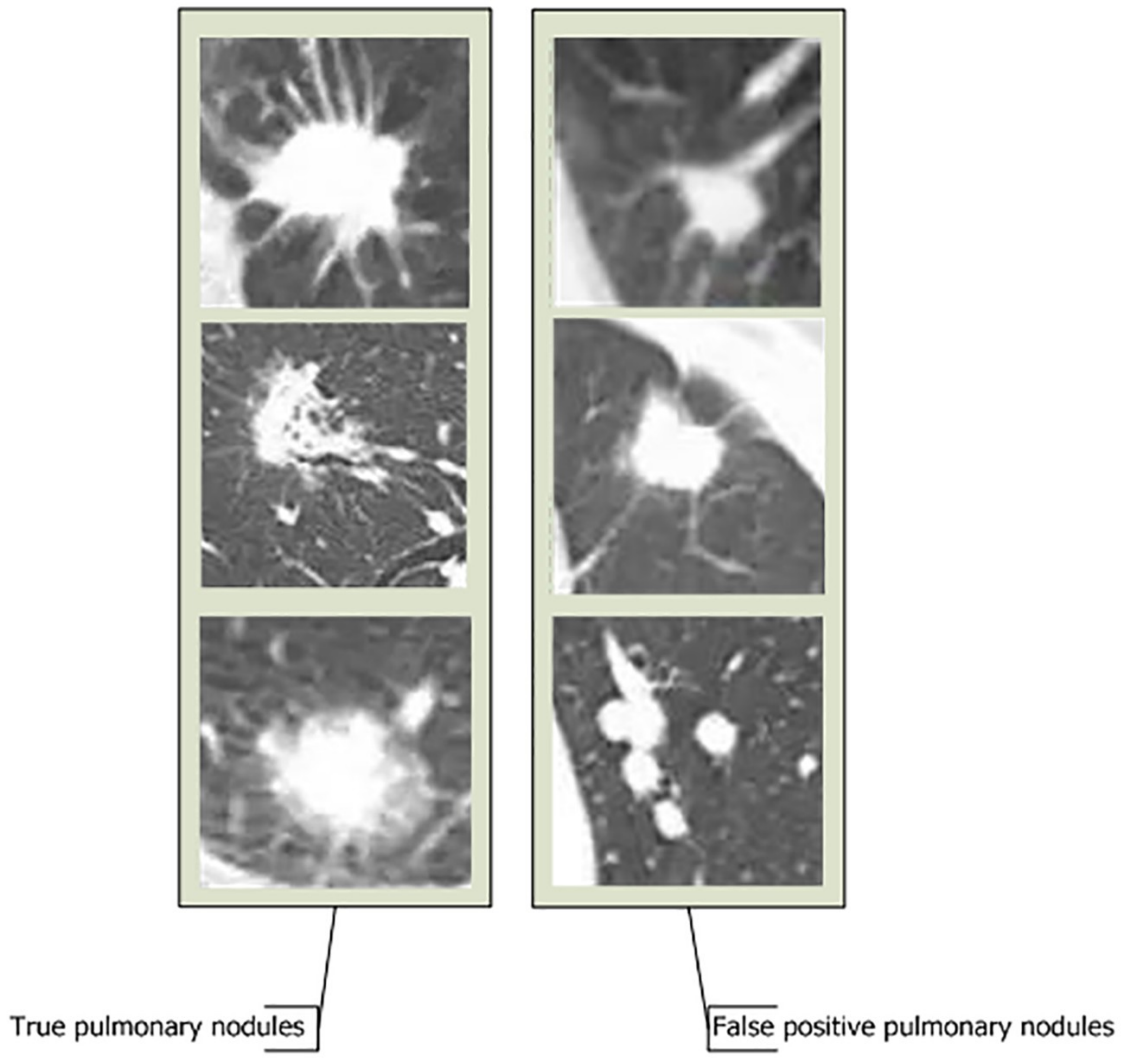
Comparison of true pulmonary nodules and false positive pulmonary nodules.

In a CNN, the convolutional and pooling layers learn the local spatial structure of the input image, while a later fully connected layer learns at a more abstract level that includes more global information. However, because a traditional neural network uses the fullyconnected layer as the final classification layer, some details and spatial features of the image will be lost when the final feature is displayed. Therefore, the outline of the nodule cannot be judged based on this approach—true nodulesare highly similar to FP nodules (as shown in Fig 2). But in some details, there are many differences between positive pulmonary nodules and false positive pulmonary nodules. For example, the margin of positive pulmonary nodules is coronal radiation, with needle-like protuberances on the margin, non-smooth margins, transparent areas in the interior and ground glass elements; while the margin of false positive nodules is smooth, without ground glass elements. Therefore, it is difficult to identify FP pulmonary nodules using a traditional CNN, and traditional CNNs achieve low accuracy scores for malignant pulmonary nodule discovery. For this reason, our team proposed a new network framework, called A-CNN, to improve the sensitivity of lung nodule detection for nodules of 5–30 mm.

## Materials and methods

### A, Materials

**Fig 3:**
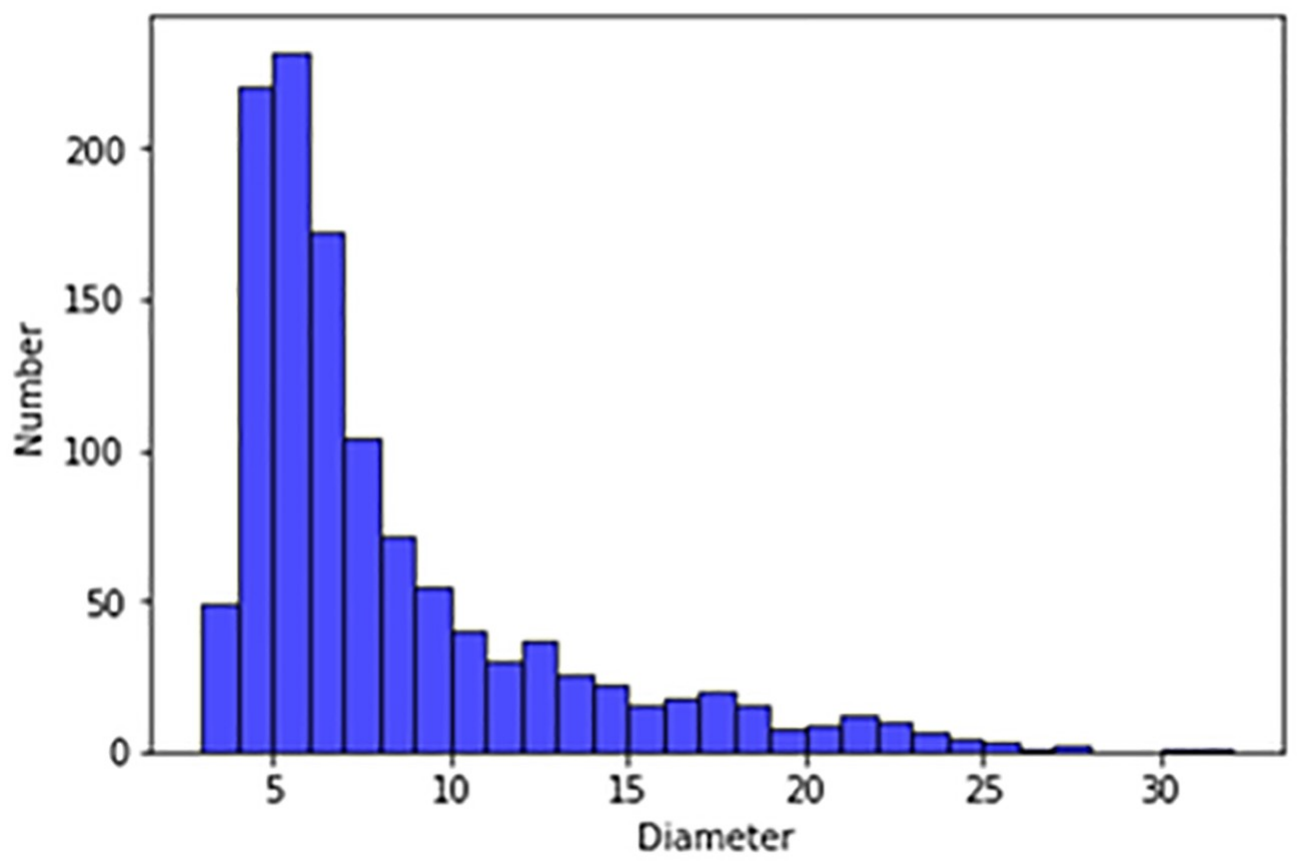
Distribution of pulmonary nodule size in the LUNA16 dataset.

**Fig 4:**
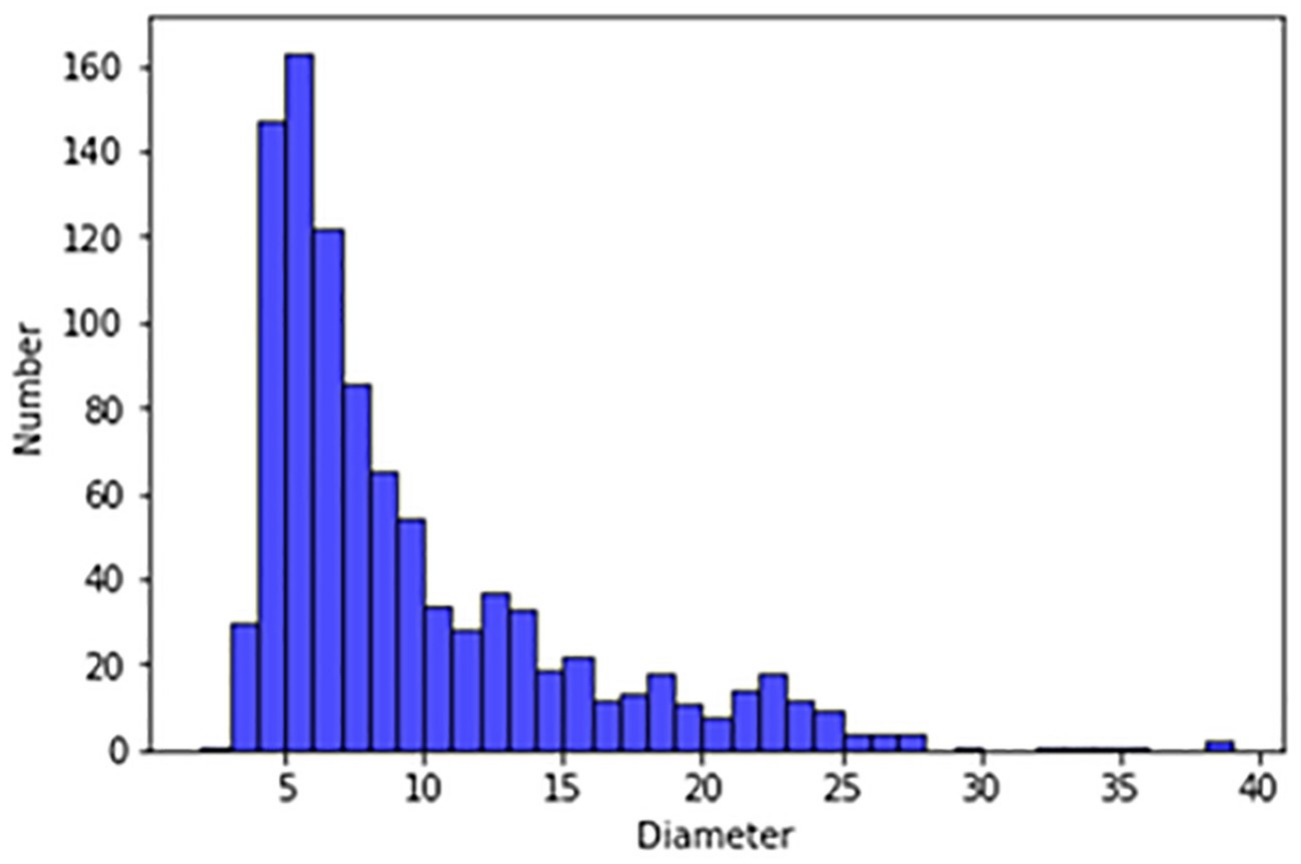
Size distribution map of pulmonary nodules in the Ali Tianchi dataset.

Our experimental dataset was derived from the publicly available LUNA16 dataset and the Ali Tianchi dataset. The LUNA16 dataset comes from a larger dataset, the Lung Image Database Consortium and Image Database Resource Initiative (LIDC/IDRI) database. In both the LUNA16 dataset and the Ali Tianchi medical dataset the slice thickness is less than 2.5 mm. Our dataset included 1,888 CT scans, each of which contained a series of axial sections of the thoracic cavity. The number of slices included in each image varied according to the scanning machine, scanning thickness, and patient. The original images are three-dimensional. Each image contain a series of axial sections of the thorax. This three-dimensional image consists of different numbers of two-dimensional images. The number of two-dimensional images can vary based on factors such as the scanning machine used and the patients. The reference standard for these datasets is that 3 of 4 radiologists accept nodes larger than 3 mm. The nodules excluded from the reference criteria (which included non nodules, nodules smaller than 3mm, and nodules accepted by only 1–2 radiologists) were considered unrelated. We excluded nodules below 5 mm, and tested only pulmonary nodules with diameters ranging from 5 mm to 30 mm for false positives. Fig 3 shows the size distribution of the pulmonary nodules in the LUNA16 dataset, and Fig 4 shows the size distribution of the pulmonary nodules in the Ali Tianchi dataset.

### B, Preprocessing

**Fig 5:**
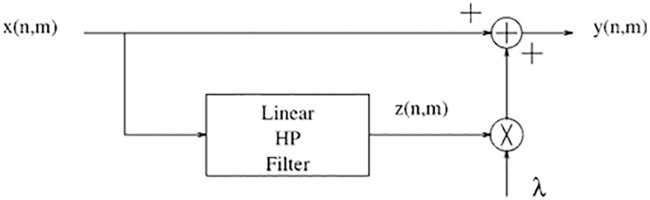
Flowchart of unsharp mask image sharpening technology.

We preprocessed the LUNA16 and Ali Tianchi datasets in three stages. First, we excluded nodules smaller than 5mmbased on the dataset labels, leaving only the nodes between 5 mm and 30 mm. Then, we segmented all the lung sections from the LUNA16 and Ali Tianchi dataset. Lung segmentation is challenging because no heterogeneity exists in the lung region. Additionally, the complex structure and uneven gray distribution of lung CT images makes it difficult to accurately segment and extract the lung parenchyma. Preprocessing lung CT images is a key step in a CADe system. Preprocessing involves removing the useless parts and redundant clutter to improve the image quality, allowing better results to be achieved to reduce FPs. For lung segmentation, the existing techniques can be divided into four categories: the threshold-based method, the variable model, and the shape or edge-based models [27]. In this paper, we use unsharp mask (UM) image sharpening technology to enhance the nodule signal in Charge eXchange Recombination Spectroscopy (CXRS). A technical flowchart is shown in Fig 5.

The formula is as follows:

y(n,m)=x(n,m)+λz(n,m), (1)

where x(n,m) is the input image, y(n,m) is the output image, and z(n,m) is a correction signalusually obtained through high-pass filtering of x. Here, λ is a scaling factor used to control the enhancement effect. In the UM algorithm, z(n,m) can be obtained by Formula (2):

z(n,m)=4x(n,m)-x(n-1,m)-x(n+1,m)-x(n,m-1)-x(n,m+1). (2)

In the second pretreatment stage, the team rotated the LUNA16 dataset and the Ali Tianchi dataset clockwise by 90°, 180°, and 270°, respectively. In the third pretreatment stage, our team divided the rotated images from the original LUNA16 dataset and the CT images from the Ali Tianchi dataset into 96×96 images and down sampled them to generate 32×32, 64×64, and 96×96 images, resulting in 326,570 training samples. In these samples, there are 18,125 pulmonary nodules.

### C, Training

**Fig 6:**
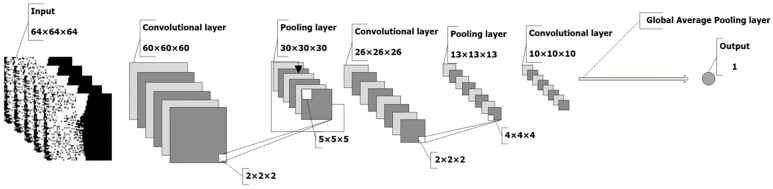
A neural network structure for detecting pseudo positive pulmonary nodules.

The CNN is composed of several convolutional layers, pooling layers, and global average pooling layers. Small 3D images can be tailored around the markers in the whole CT image; these small 3D images correspond directly to the nodule labels. By learning these features, a neural network is trained to detect pulmonary nodules, evaluate the malignance degree of nodules, and predict the possibility of cancer in patients. During prediction, the neural network traverses the entire CT image and judges the possibility that malignant information occurs in each position of a sliding window. The above process is illustrated in Fig 6.

The activation function is very important during CNN calculations. Traditional neural networks usually use the rectified linear unit (ReLU) function as an activation function because compared with other linear functions, ReLU is more expressive, especially in deep networks. For nonlinear functions, ReLU does not have the vanishing gradient problem because the gradient of nonnegative intervals is constant, and the convergence rate of the model is stably maintained. The problem of gradient vanishing is that when the gradient becomes less than 1, the error between the predicted value and the real value will decay once per layer of propagation. This phenomenon is particularly obvious when a sigmoid function is used as the activation function in a deep model and will lead to stagnation during convergence. The mathematical expression of the ReLU function is shown in Formula (3):

$$f(x)=\left\{ \begin{array}{c} 0,x\leq 0\\ x,x\geq 0 \end{array}.\right.$$ (3)

When the input value to ReLU is negative, the output and first derivative are always 0. This causes the neurons to fail to update the parameters, and this phenomenon is called a Dead Neuron. To address the shortcomings of the ReLU function, our team used a new activation function—Random Translation ReLU (RT-ReLU) [28]as the activation function of our A-CNN fusion neural network. Cao et al. made full use of the input distribution of the nonlinear activation function and proposed the random translation nonlinear activation function for deep CNNs. During training, the nonlinear activation function is randomly converted from the offset of the Gaussian distribution, while during testing, a nonlinear activation with zero offset is used. Based on the method proposed by Cao et al., the input distribution of nonlinear activation is relatively decentralized, meaning that the acquired CNN is robust to small fluctuations of the nonlinear activation input. Thus, RT-ReLU is conducive to reducing over fitting of the neural network. The mathematical expression of RT-ReLU is shown in Formula (4):

$$y_{i}=\left\{ \begin{array}{c} x_{i}+a_{i},\;\text{if}\;x_{i}+a_{i}>0\\ 0,\;\text{if}\;x_{i}+a_{i}\leq 0 \end{array}\right..$$ (4)

**Fig 7:**
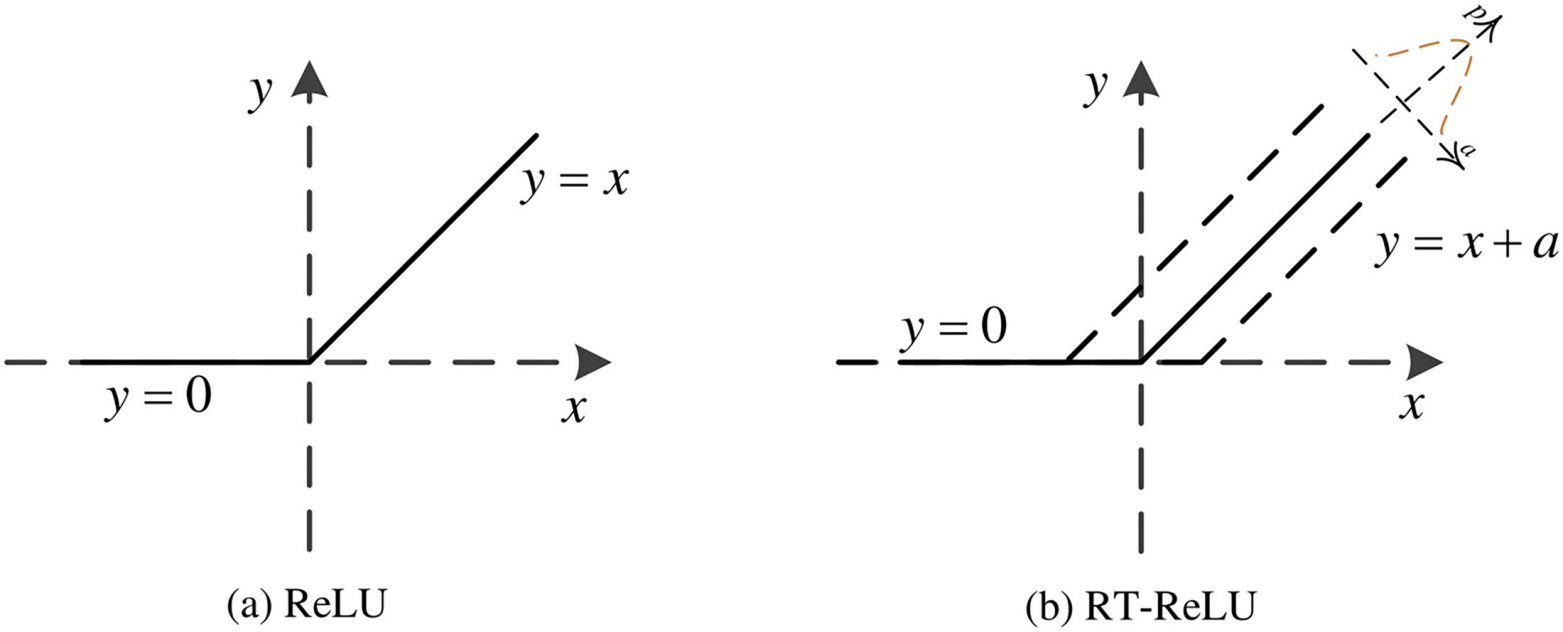
Contrast graph of ReLU function and RT-ReLU function.

The functional models of the ReLU and RT-ReLU functions are shown in Fig 7:

A single CNN has a limited learning ability; therefore, while a single CNN may be able to identify all the basic features necessary to distinguish nodules from different types of nonnodular structures, using multiple different CNNs can distinguish more nonnodules. Therefore, our team proposed the A-CNN to detect pulmonary nodules.

**Fig 8:**
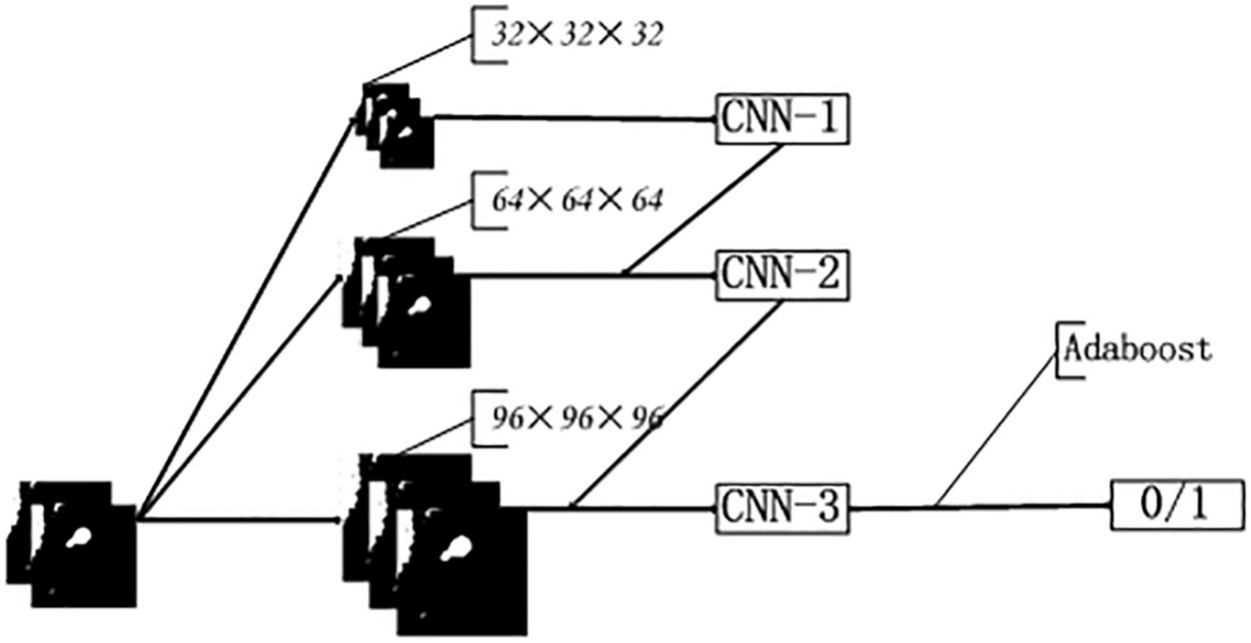
The structure of the neural network.

We constructed three types of 3D-CNN structures for different input scales, namely, CNN-1, CNN-2, and CNN-3. CNN-1 used 32×32×32 CT segmentation images as input, CNN-2 used 64×64×64 images, and CNN-3 used 96×96×96 images as input. The structures of these CNNs are shown in Table 1. The structure of the proposed A-CNN is shown in Fig 8. The results of the three models are fused using an AdaBoost classifier.

**Table 1:** Single CNN structure table.

Layer		Type	Input	Kernel	Stride	Pad	Output
CNN-1	0	Input	32×32×32	N/A	N/A	N/A	32×32×32
	1	Convolutional	32×32×32	5×5×5	1	0	20×32×32×32
	2	Max pooling	20×32×32×32	2×2×2	2	0	20×14×14×14
	3	Convolutional	20×14×14×14	5×5×5	1	0	50×10×10×10
	4	Global Average Pooling					
CNN-2	0	Input	64×64×64	N/A	N/A	N/A	64×64×64
	1	Convolutional	64×64×64	5×5×5	1	0	20×60×60×60
	2	Max pooling	20×60×60×60	2×2×2	2	0	20×30×30×30
	3	Convolutional	20×30×30×30	5×5×5	1	0	50×26×26×26
	4	Max pooling	50×26×26×26	2×2×2	2	0	50×13×13×13
	5	Convolutional	50×13×13×13	4×4×4	1	0	100×10×10×10
	6	Global Average Pooling					
CNN-3	0	Input	96×96×96	N/A	N/A	N/A	96×96×96
	1	Convolutional	96×96×96	5×5×5	1	0	20×92×92×92
	2	Max pooling	20×92×92×92	2×2×2	2	0	20×46×46×46
	3	Convolutional	20×46×46×46	5×5×5	1	0	50×42×42×42
	4	Max pooling	50×42×42×42	2×2×2	2	0	50×21×21×21
	5	Convolutional	50×21×21×21	5×5×5	1	0	100×17×17×17
	6	Max pooling	100×17×17×17	2×2×2	2	0	100×9×9×9
	7	Convolutional	100×9×9×9	5×5×5	1	0	100×5×5×5
	8	Global Average Pooling					

Different CNN architectures can process different input images and different scales of CT images. The A-CNN model proposed by our team combines the advantages of CNN-1, CNN-2, and CNN-3. We conducted a random 10-fold validation test to verify our algorithm and demonstrate its high robustness.

### D, AdaBoost classifier reduces false positive nodules

The principle underlying A-CNN is shown in Fig 8. The boosting family of algorithms can upgrade weak learners to strong learners. The core idea of boosting is to train different classifiers (weak classifiers) using the same training set and then create an ensemble of those trained weak classifiers to form a stronger final classifier (strong classifier). The algorithm itself changes the data distribution by determining the weight of each sample based on whether the classification of each sample in each training set is correct, including the accuracy of the last overall classification. The new dataset with modified weights is sent to the individual classifiers for training. Finally, the classifiers obtained from each training operation are fused to form the final classifier.

The working approach to A-CNN is to train CNN-1 from the training set with the initial weights, update the weights of the training samples according to the learning error rate performance of CNN-1, make the weights of the training samples with high learning error rates by CNN-1 become higher to pay more attention to the points with high learning error rates in the subsequent CNN-2. Then, CNN-2 is trained based on the training set after weight adjustment. The points with high error rates in CNN-2 are treated similarly so that they will receive more attention in the subsequent CNN-3. Then, CNN-3 is trained based on the training set after the weight adjustment. This operation is repeated until CNN-1, CNN-2 and CNN-3 are eventually integrated through a set strategy to form the final A-CNN. The specific algorithm operation is as follows:

Recognition of pulmonary nodules is a two-class problem. The input training set is shown in Formula (5):

$$T=\{(x_{1},y_{1}),(x_{2},y_{2}),\ldots (x_{m},y_{m})\},$$ (5)

whose output is {-1, +1}.

Initialize the training set weight as shown in Formula (6):

$$D(1)=(\omega_{11},\omega_{12}\ldots \omega_{1m});\;\omega_{1i}=\frac{1}{m};\;i=1,2\ldots m,$$ (6)

For k = 1,2…K:

The weighted $D_{k}$ training set is used to train the data, and the weak classifier $G_{k}(x)$ is obtained.

The classification error rate of $G_{k}(x)$ is calculated as shown in Formula (7):

$$e_{k}=P(G_{k}(x_{i})\neq y_{i})=\sum_{i=1}^{m}\omega_{ki}I(G_{k}(x_{i})\neq y_{i}),$$ (7)

The coefficients of each weak classifier are calculated as shown in Formula (8):

$$\alpha_{k}=\frac{1}{2}log\frac{1-e_{k}}{e_{k}},$$ (8)

where $e_{k}$ represents the classification error rate, and $\alpha_{k}$ represents the weight coefficient of the weak classifier. As the above formula shows, when the classification error rate $e_{k}$ is larger, the corresponding weak classifier weight coefficient $\alpha_{k}$ is smaller; in other words, the smaller the error rate is, the larger the weight coefficient of the weak classifier is.

Update the sample weight D. Assuming that the sample set weight coefficient of the k th weak classifier is $D(K)=(\omega_{k1},\omega_{k2}\ldots \omega_{km})$, the corresponding sample set weight coefficient of the (k+1)th weak classifier is shown in formula (9):

$$\omega_{k+1},i=\frac{\omega_{ki}}{Z_{K}}exp(-\alpha_{k}y_{i}G_{k}(x_{i}))\;i=1,2\ldots m,$$ (9)

where $Z_{k}$ is the normalization factor, as shown in Formula (10):

$$Z_{k}=\sum_{i=1}^{m}\omega_{ki}exp(-\alpha_{k}y_{i}G_{k}(x_{i})),$$ (10)

From the formula for $\omega_{k+1},i$, it can be seen that when the classification of the first sample is incorrect, $y_{i}G_{k}(x_{i})<0$, which leads to an increased weight of the sample in the k+1 weak classifier. Similarly, a decrease in the weight occurs in the k+1 weak classifier when the classification is correct.

The final classifier is constructed as shown in Formula (11):

$$f(x)=sign(\sum_{k=1}^{K}\alpha_{k}G_{k}(x)),$$ (11)

### E, Evaluation criterion

Finally, we validated the new detection method of pulmonary nodules with this new neural network. Our team uses metrics commonly used in CAD systems and widely used for performance analyses of image processing systems [29].

Sensitivity (SEN), also known as the true positive rate, is expressed by dividing the number of true positives by the total number of positive cases, as shown in Formula (12):

$$\text{SEN}=\frac{TP}{TP+FN},$$ (12)

where *TP* denotes true positive, *FN* denotes false positives.

In the two-class problem of false positive reduction, we use thefree-responsereceiver operating characteristic (FROC) curve to evaluate our A-CNN model. The FROC curve is a coordinate map composed of the average number of false positives per scan as the horizontal axis, the sensitivity as the vertical axis, and the subject’s specific peak. Under excitation conditions, the curves drawn from different results are obtained by different criteria. Traditional diagnostic test evaluation methods have a common feature. The test results must be divided into two categories and then statistically analyzed. The evaluation method of the FROC curve is different from the traditional evaluation method. It does not require this restriction; instead, it allows the evaluation of multiple anomalies on an image based on the actual situation, making the FROC curve evaluation method more applicable. Therefore, we adopted the FROC curve as the evaluation criterion.

## Experiment and results

This section presents and discusses the results obtained from the detection of pulmonary nodules from CT scans. We implemented the proposed neural network A-CNN to detect pulmonary nodules in the Python programming language on TensorFlow using the Keras deep learning library. We executed the model on a computer equipped with an Intel I7-8700 processor, 16 GB RAM, an Nvidia GeFrand GTX 1080Ti, and a Windows 10 operating system.

We preprocessed the LUNA16 dataset and the lung nodule slices from the Ali Tianchi dataset and obtained 326,570 slices. To test the effective detection of the new A-CNN model, we randomly divided the processed datasets into three groups: training, verification, and testing. The training set contains 266,570 slices, the verification set contains 30,000 slices and the test set contains 30,000 slices. Among these, the training set contains 14,674 nodules, the test set contains 1,795 nodules, and the verification set contains 1,656 nodules.

We trained the CNN-1, CNN-2, CNN-3, and the fusion neural network A-CNN; then, we verified and tested them using the same verification and test set to achieve a fair comparison between each neural network. Based on the cross-entropy loss function, we used stochastic gradient descent to supervise the training of each neural network, where the difference between the expected and current output of the network is continuously reduced by adjusting the weight. The weights were initialized to a standard value and updated by a standard back propagation algorithm for 30 epochs. The weights were attenuated to 0.0005, and the learning rate was set to 0.001.

**Fig 9:**
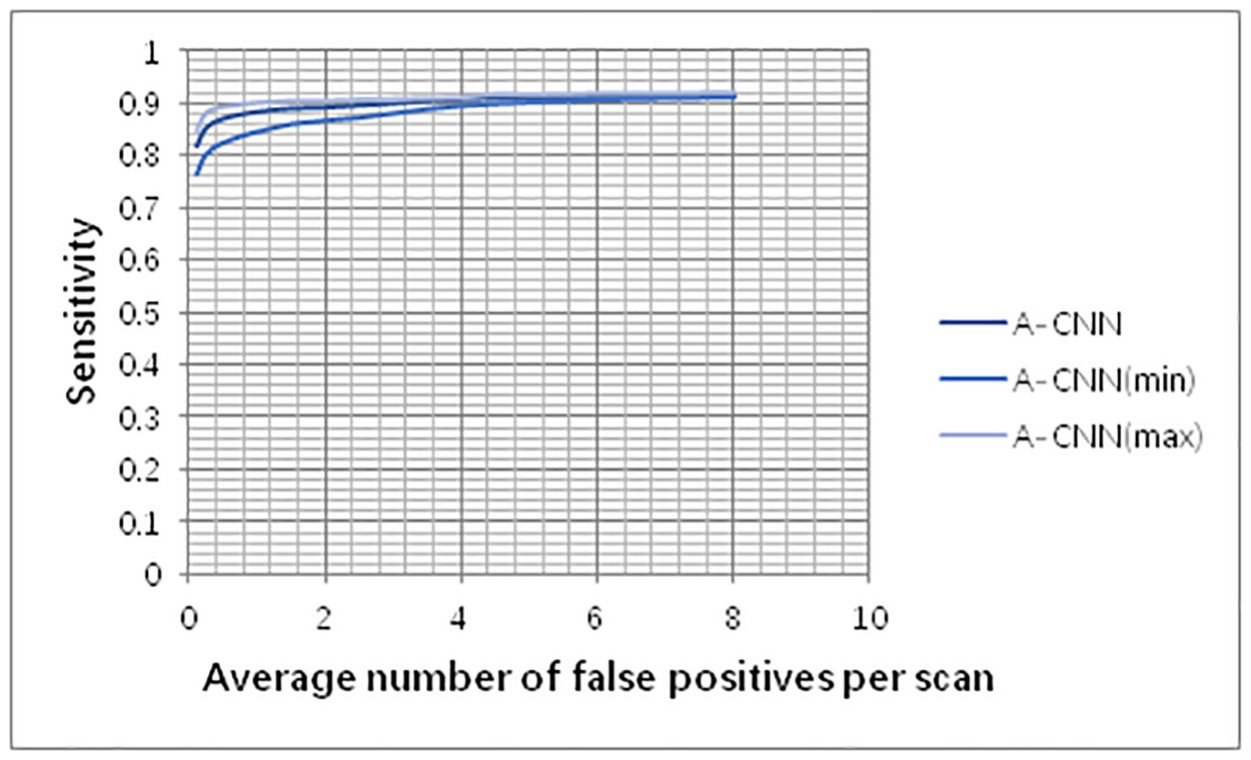
FROC curve for pulmonary nodule detection.

We tested the false positive reduction performance of the fusion A-CNN on the LUNA16 and Ali Tianchi datasets. The results show that when the average number of false positives per scan is 0.125 FPs/scan, 0.25 FPs/scan, 0.5 FPs/scan, 1 FPs/scan, 2 FPs/scan, 4 FPs/scan, and 8 FPs/scan, the model achieves sensitivity values of 0.817, 0.851, 0.869, 0.883, 0.891, 0.907, and 0.914, respectively. We conducted a random 10-fold validation test to verify the robustness of the algorithm. As shown in Fig 9, A-CNN has high robustness.

**Fig 10:**
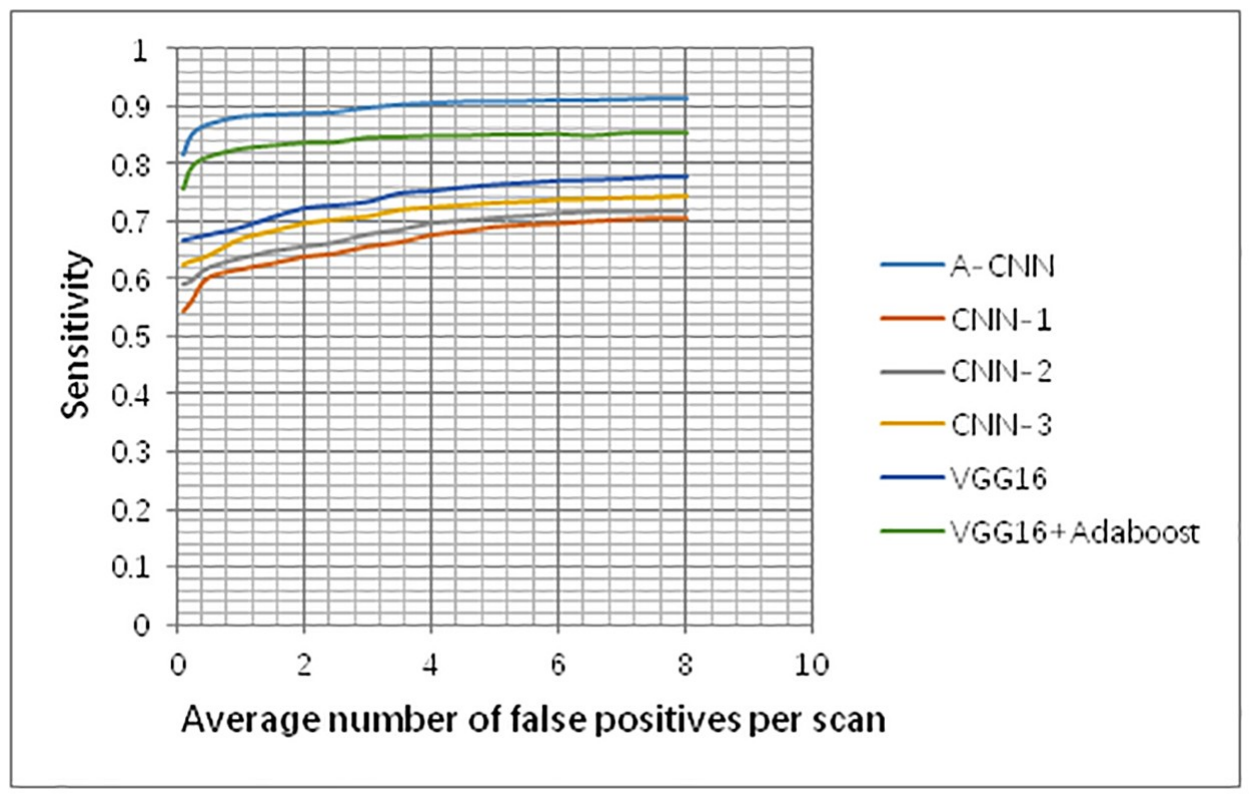
Performance comparison of A-CNN with CNN-1, CNN-2 and CNN-3.

FROC curves using the three individual CNNs and A-CNN are shown in Fig 10. When the average number of false positives per scan is 0.125 FPs/scan and 0.25 FPs/scan, the sensitivity of A-CNN reaches 81.7% and 85.1%, while the sensitivities of the other four single CNNs can only be up to 62.3% and 62.9%, respectively (as shown in Fig 10). These results demonstrate that A-CNN is significantly better than are the individual CNN-1, CNN-2, CNN-3and VGG16 models.

The AdaBoost classifier is suitable for fusing multiple weak classifiers and it performs well with weak classifiers. Therefore, our team compared CNN-1, CNN-2, and CNN-3 with the VGG16 neural network. As Fig 10 shows, VGG16 performs better than the other three neural networks. And our team compared A-CNN with VGG16+AdaBoost neural network, A-CNN showed better performance. Therefore, the AdaBoost classifier is suitable for use in A-CNN in this paper.

In this study, we used UM image sharpening technology to enhance the nodule signals in CXRS. Therefore, as verification, we input the original images and the preprocessed images into A-CNN and compared the average sensitivity between the two types of input, as shown in Table 2. The results show that UM image processing can slightly increase the sensitivity of nodule detection. Although the average sensitivity difference before and after UM image processing is only 0.015, this result shows that UM processing of CT image is beneficial to improve the overall detection sensitivity.

**Table 2:** Comparison of average sensitivity between input of original images and those after image sharpening.

Input	Average result
Original graph	0.876
Unsharp Mask	0.861

The relevant works cited in this paper do not provide their training, validation, or the test cases used, which makes it difficult to compare the experimental results reported in this paper to those of other related works. The only information the other works provided was the database used; consequently, we were unable to perform a rigorous methodological evaluation of other works, and are able to compare the FROC curve of pulmonary nodules only with the results of the known literature.

**Fig 11:**
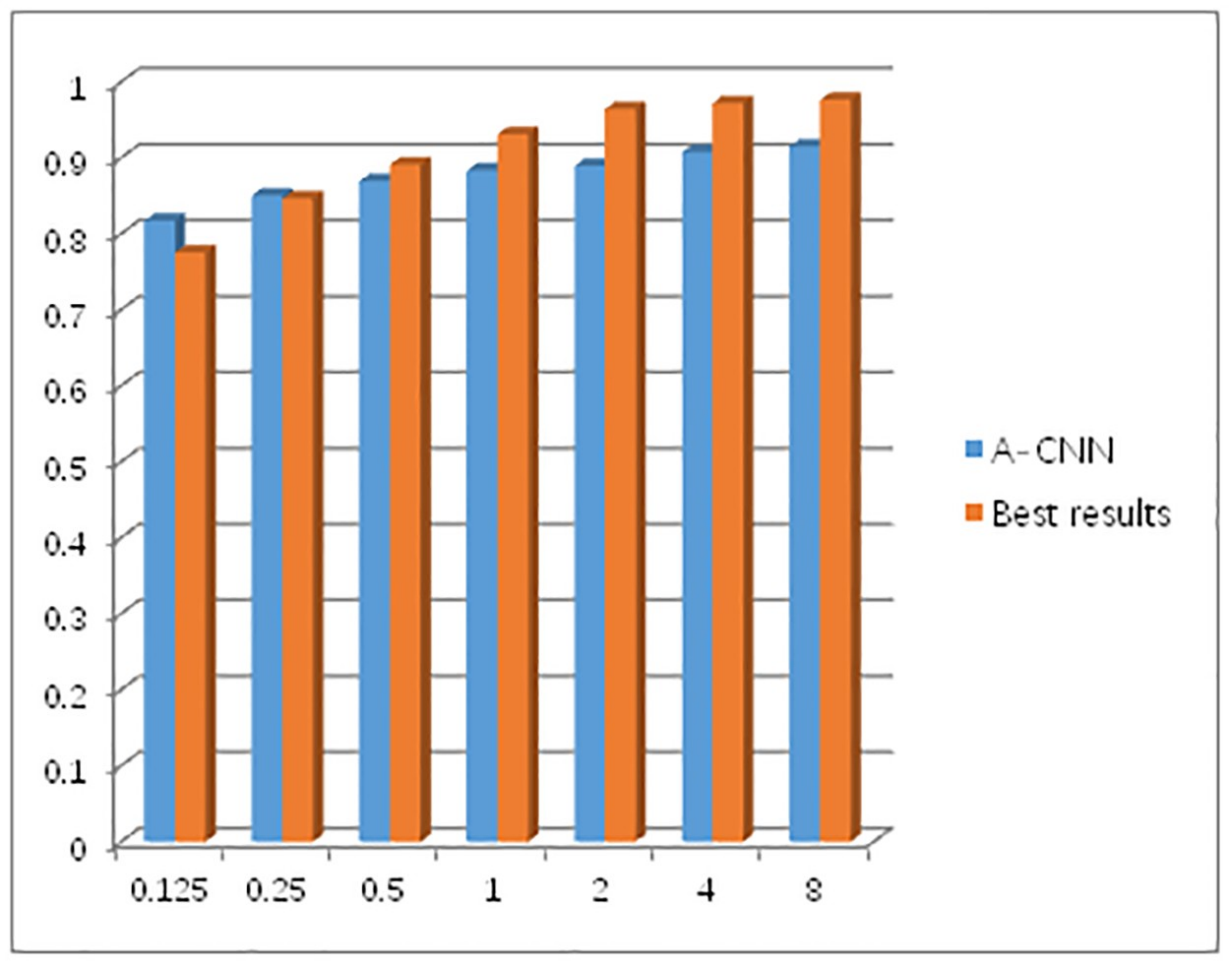
A comparison of pulmonary nodules detection results between A-CNN and the best results of other methods.

Table 3 compares the false positive results of the LUNA16 challenge between our team and those of other teams. We compare lung nodule test results only with other published test methods. Each team used a deep CNN to exclude false positive results. Two of the nine teams are based on 2D-CNN architectures, while seven are based on 3D-CNN architectures. In this study, we used a 3D-CNN framework [30] to detect pulmonary nodules. 3D-CNNsare conducive to the analysis and detection of pulmonary nodules from a stereoscopic perspective, and their pulmonary nodules detections are more sensitive than those of 2D-CNNs. It is worth noting that when the average number of false positives per scan is 0.125FPs/scan and 0.25FPs/scan, the sensitivity of A-CNN can reach 81.7% and 85.1%, respectively, which represent the best performances. Fig 11 clearly shows the sensitivity of A-CNN for the detection of pulmonary nodules compared to the best results by other teams.

**Table 3:** Sensitivity of pulmonary nodules compared with other groups.

Team	CNN	0.125	0.25	0.5	1	2	4	8
Xie[19]	2D	0.734	0.744	0.763	0.796	0.824	0.832	0.834
RESNET	2D	0.675	0.745	0.813	0.879	0.903	0.939	0.948
MEDICAI	3D	0.744	0.802	0.847	0.881	0.901	0.927	0.928
Aidence	3D	0.747	0.801	0.834	0.872	0.922	0.963	**0.976**
3DCNN_NDET	3D	0.733	0.809	0.872	0.918	0.937	0.960	0.964
CASED	3D	0.775	0.835	0.869	0.903	0.928	0.953	0.965
iDST-VCT	3D	0.745	0.827	0.889	**0.931**	**0.964**	**0.972**	0.975
qfpxfd	3D	0.763	0.846	**0.891**	0.848	0.919	0.943	0.949
**A-CNN**	3D	**0.817**	**0.851**	0.869	0.883	0.891	0.907	0.914

Compared to other methods, A-CNN achieved highsensitivity and better detection results for pulmonary nodules. For a CAD system, sensitivity is a key measure because it reflects the performance of the model in correctly classifying nodules and achieving more convenient medical monitoring.

## Conclusions

In this study, three CNN frameworks were establishedand then fused into a new A-CNN model to detect pulmonary nodules. The A-CNN network model proposed by our team was validated on the LUNA16 dataset, where it achieved sensitivity scores of 81.7% and 85.1% whenthe average false positives number per scan was 0.125 FPs/scan and 0.25 FPs/scan, respectively. These scores were 5.4% and 0.5% higher than those of the current optimal algorithm; thus, our A-CNN model can improve the efficiency of radiological analysis of CT.
